# VISTA: A Target to Manage the Innate Cytokine Storm

**DOI:** 10.3389/fimmu.2020.595950

**Published:** 2021-02-11

**Authors:** Mohamed A. ElTanbouly, Yanding Zhao, Evelien Schaafsma, Christopher M. Burns, Rodwell Mabaera, Chao Cheng, Randolph J. Noelle

**Affiliations:** ^1^ Department of Microbiology and Immunology, Norris Cotton Cancer Center, Geisel School of Medicine at Dartmouth, Lebanon, NH, United States; ^2^ Department of Medicine, Baylor College of Medicine, Houston, TX, United States; ^3^ Department of Molecular and Systems Biology, Geisel School of Medicine at Dartmouth, Hanover, NH, United States; ^4^ Department of Medicine, Norris Cotton Cancer Center, Lebanon, NH, United States; ^5^ Dan L Duncan Comprehensive Cancer Center, Baylor College of Medicine, Houston, TX, United States; ^6^ Institute for Clinical and Translational Research, Baylor College of Medicine, Houston, TX, United States

**Keywords:** vista, cytokine storm, myeloid, immune checkpoint, agonistic antibodies, immunosuppression, monocyte reprogramming

## Abstract

In recent years, the success of immunotherapy targeting immunoregulatory receptors (immune checkpoints) in cancer have generated enthusiastic support to target these receptors in a wide range of other immune related diseases. While the overwhelming focus has been on blockade of these inhibitory pathways to augment immunity, agonistic triggering *via* these receptors offers the promise of dampening pathogenic inflammatory responses. V-domain Ig suppressor of T cell activation (VISTA) has emerged as an immunoregulatory receptor with constitutive expression on both the T cell and myeloid compartments, and whose agonistic targeting has proven a unique avenue relative to other checkpoint pathways to suppress pathologies mediated by the innate arm of the immune system. VISTA agonistic targeting profoundly changes the phenotype of human monocytes towards an anti-inflammatory cell state, as highlighted by striking suppression of the canonical markers CD14 and Fcγr3a (CD16), and the almost complete suppression of both the interferon I (IFN-I) and antigen presentation pathways. The insights from these very recent studies highlight the impact of VISTA agonistic targeting of myeloid cells, and its potential therapeutic implications in the settings of hyperinflammatory responses such as cytokine storms, driven by dysregulated immune responses to viral infections (with a focus on COVID-19) and autoimmune diseases. Collectively, these findings suggest that the VISTA pathway plays a conserved, non-redundant role in myeloid cell function.

## Introduction

The immunoglobulin superfamily (IgSF) includes over 750 members (1, 2); at least 300 of which play an immunoregulatory inhibitory role in immune cell activation and function (3, 4). These inhibitory receptor checkpoints have proven crucial for the maintenance of balanced immune responses and managing the threshold for tolerance and prevention of immunopathology (5). Immunoregulatory receptors can be broadly categorized into either receptors that (1) control immune cell homeostasis and negatively regulate activation, or (2) receptors mediating negative feedback regulation of activation. The most dominant examples of immune checkpoints are CTLA-4 and PD-1 whose roles in immune tolerance and immunotherapeutic benefits have been extensively investigated. However, this class of inhibitory receptors along with every other immune checkpoint in clinical development (LAG-3, TIGIT, TIM-3 and others) all belong to the second class of immunoregulators; those that serve a negative feedback mechanism to inhibit dysregulated immune responses and normalize immunity. VISTA is an immunoregulatory receptor belonging to the first class of threshold homeostatic receptors, wherein it is expressed at steady state on both T cells and myeloid cells and whose expression remains high and is downregulated depending on the activation stimulus. This is in contrast to the negative feedback immune checkpoints (CTLA-4 and others) which are expressed after immune cell activation and mediate different inhibitory mechanisms to restrain further activation or responses. Of equal importance, the majority of immunoregulatory receptors of promising therapeutic relevance are expressed on T cells, and therefore set to negatively regulate T cell responses. However, a significant contribution to immunopathology is mediated by myeloid cell hyperinflammatory responses. Particularly, in the setting of cytokine storms which occur in viral infections, inflammatory diseases and allogeneic responses, monocytes and macrophages are the major producers of inflammatory cytokines that contribute to tissue damage and lethality (6–8). Therefore, there is an unmet need for the development of immunoregulatory targeting strategies that selectively inhibit innate immune responses at various stages of activation. VISTA emerges as an advantageous target here given its high constitutive expression on myeloid cells [primarily monocytes, macrophages and neutrophils] as well as its intrinsic inhibitory role on these cell types. Of equal value is the ability to target VISTA with agonistic antibodies to drive negative regulation and to inhibit inflammation and augment tolerance. Therefore, VISTA represents a major target on both T cells and myeloid cells with agonistic antibodies. Other reviews and studies have detailed the current knowledge on VISTA biology on T cells, as well as the potential of its blockade in the settings of cancer (9, 10). This review will primarily focus on very recent mechanistic insights and findings from human and mouse systems elucidating a potential role for VISTA in management of innate inflammation. This focus is particularly relevant given the recent series of reports on the pathogenic impact of heightened innate inflammation in COVID-19.

## Methods 

### Single Cell RNA Sequencing and Normalization

Droplet-based 5′ end single-cell RNA sequencing (scRNAseq) was performed using 10x Genomics platform. The libraries were prepared using the Chromium Single Cell 5’ Reagent kit according to the manufacturer’s protocol (10x Genomics, CA, USA).

Barcode processing and transcript counts were conducted using The Cell Ranger Single-Cell Software Suite (10x Genomics) after alignment to the human GRCh38 reference genome with default parameters. Then, the Seurat R package was applied to filter out low-quality cells, normalize gene expression profiles and cluster cells. Cells expressing >10% mitochondrial gene counts or expressing less than 500 genes were discarded using *FilterCells* function. *NormalizeData* function was applied to normalize and log transform raw counts for each cell based on its library size.

### Single Cell Unsupervised Clustering

The normalized expression matrices of CD14^+^ human monocytes were processed by filtering the non-expressed genes separately. The unsupervised clustering was applied in each dataset as follows: 1. Top 2,000 variant genes were selected and used as the input for Principal Component Analysis (PCA) to reflect the major biological variation in the data. 2. Top 15 PCs were chosen for UMAP dimension reduction by *RunUMAP* function and unsupervised clustering. In specific, *FindClusters* function was used to cluster the cells. 3. After the cell clusters were determined, marker genes for each cluster were identified by the *FindAllMarkers* function with the default parameter. The biological annotation of each cluster was further described by the markers genes function reported in the literature.

### Pathway Enrichment Analysis

The differentially expressed genes between Anti-VISTA (803) and hIgG treated CD14+ human monocytes were ranked based on the average log2-fold change. To annotate the pathways that were involved in the differentially expressed genes, pathway gene sets were downloaded from the C2 category of the Molecular Signatures Database (MSigDB v6.2) database (11). The preranked Gene set enrichment analysis (GSEA) software was used to calculate the enrichment of each pathway in the genes that are most informative in each gene list.

### RNA-Seq Alignment for Anti-VISTA (803) and hIgG Treated CD14+ Human Monocytes Total RNA-Seq

Sequencing was performed on a NextSeq 500 (Illumina) instrument to obtain an average of raw 100bp single end reads per sample. Raw.bcl files were demultiplexed using the Illumina bcl2fastq2 pipeline. The quality of the fastq files was examined with the *FastQC* software (http://www.bioinformatics.babraham.ac.uk/projects/fastqc). Raw fastq files were trimmed using the software *Trimmomatic* by setting the parameter “SLIDINGWINDOW: 4:15 LEADING: 3 TRAILING: 3 MINLEN: 36”. The trimmed fastq files were than aligned to the human GRCh38 reference genome and normalized to obtain Transcripts Per Kilobase Million (TPM) for each RNA-seq sample using the software *Salmon* with the parameter “-l A” (12). DEseq2 package (13) was used to identify the differential expressed genes between Anti-VISTA (803) and hIgG treated CD14+ human monocytes based on the raw counts of the gene expression.

### Monocyte Isolation and Treatments

CD14^+^ monocytes were isolated using human CD14 microbeads (Miltenyi Biotec, 130-050-201) and purity was confirmed by flow cytometry. The cells were then incubated in complete RPMI-1640 media at a density of 1 x 10^6^ cells/ml in 6-well plates and treated with either anti-VISTA agonist (clone 803) or hIgG2 isotype control antibody at 10 ug/ml for 24 h. Multiplex analysis was performed to determine CXCL10 levels.

### Flow Cytometry

Twenty four hours after treatment, monocytes were stained with CD14 (Biolegend, clone M5E2) and CD16 antibodies (Biolegend, 3G8). For all staining, cells are incubated in FACS buffer (PBS with 0.5% BSA and 0.1% sodium azide) on ice for 20 min followed by two washes in PBS and samples were run on a MACSQuant.

## Targeting VISTA With Agonists Suppresses Multiple Inflammatory Diseases and Autoimmune Pathologies

There is accumulating evidence that targeting VISTA with agonistic antibodies can exert profound negative immunomodulatory effects with several very recent studies shedding light on novel insights from multiple inflammatory models in mice. Early work demonstrated the negative regulatory role of VISTA by the fact that aged VISTA-deficient mice (8 to 9-months of age) showed signs of chronic inflammation, highlighted by splenomegaly, enhanced cell activation markers, accumulation of inflammatory chemokines and cytokines as well as enhanced immune cell infiltration in nonlymphoid tissues (14). Heightened susceptibility to autoimmunity, was evident upon interbreeding of VISTA^-/-^ onto the Sle1.Sle3 background with strikingly enhanced lupus nephritis (15). Similarly, anti-VISTA antagonist phenocopied the impact of VISTA deficiency in exacerbating murine lupus (16). However, unlike the anti-VISTA antagonists, VISTA agonists suppressed disease in the Fas^lpr^ model of cutaneous and systemic lupus, psoriasis and other inflammatory disorders (10, 17, 18).

Most of the interest in VISTA function has focused on its immunoregulatory role on CD4^+^ T cells since targeting VISTA on T cells elicits several immunoregulatory phenotypes. One of the most striking effects of VISTA on T cell biology is evidenced by the immunosuppressive impact of anti-VISTA agonists in acute Graft-versus-Host-disease (GVHD). These studies demonstrate VISTA agonistic targeting at the time of donor T cell transfer completely prevents disease (10, 19, 20). In this setting, we showed that selective targeting by VISTA agonists [but not antagonists] to donor T cells inhibited GVHD *via* specific peripheral deletion of donor alloreactive T cells, and this mechanism was T-cell intrinsic (10). These studies suggested that strong signaling through TCR and VISTA resulted in T cell apoptosis, and offer provocative strategies to induce antigen-specific T cell tolerance. Additional insights into the role of VISTA in T cell fate comes from studies on the conditional deletion of VISTA on naïve T cells which leads to the accumulation of CD44^hi^ memory-phenotype CD4^+^ T cells with a T-bet^hi^ profile, suggesting a potential role of VISTA in suppressing Th1 and memory-phenotype T cell differentiation (10). We also generated an immune signature from VISTA-deficient naïve T cells which showed that peripheral T cells from systemic lupus erythematosus (SLE) and from rheumatoid arthritis (RA) patients presented a higher VISTA-loss immune signature compared to healthy T cells (10). Collectively, these findings support a central regulatory role for VISTA in controlling T cell survival and suppression of pathogenic T cell self-reactivity. They also infer that VISTA may be a potential diagnostic biomarker in these inflammatory diseases. More recent insights reveal a global upregulation of the interferon-I pathway in VISTA^-/-^ T cells which is known to upregulate T-bet expression (10). These findings are in agreement with another study showing that decreased VISTA expression facilitates Th1 and Th17 T cell differentiation (21). In a model of experimental asthma, VISTA deficiency and blockade exacerbated Th2 responses and type II immunity (11, 22), whereas an anti-VISTA agonistic antibody reduces disease severity and suppressed lung inflammation (22). There are reported roles of VISTA deficiency in several other inflammatory diseases including psoriasis, transplant rejection, acute hepatitis, and indicate a potential value of VISTA agonists in these immunopathologies (12, 23, 24).

## VISTA Is a Pleiotropic Myeloid Cell Checkpoint

**Figure 1:**
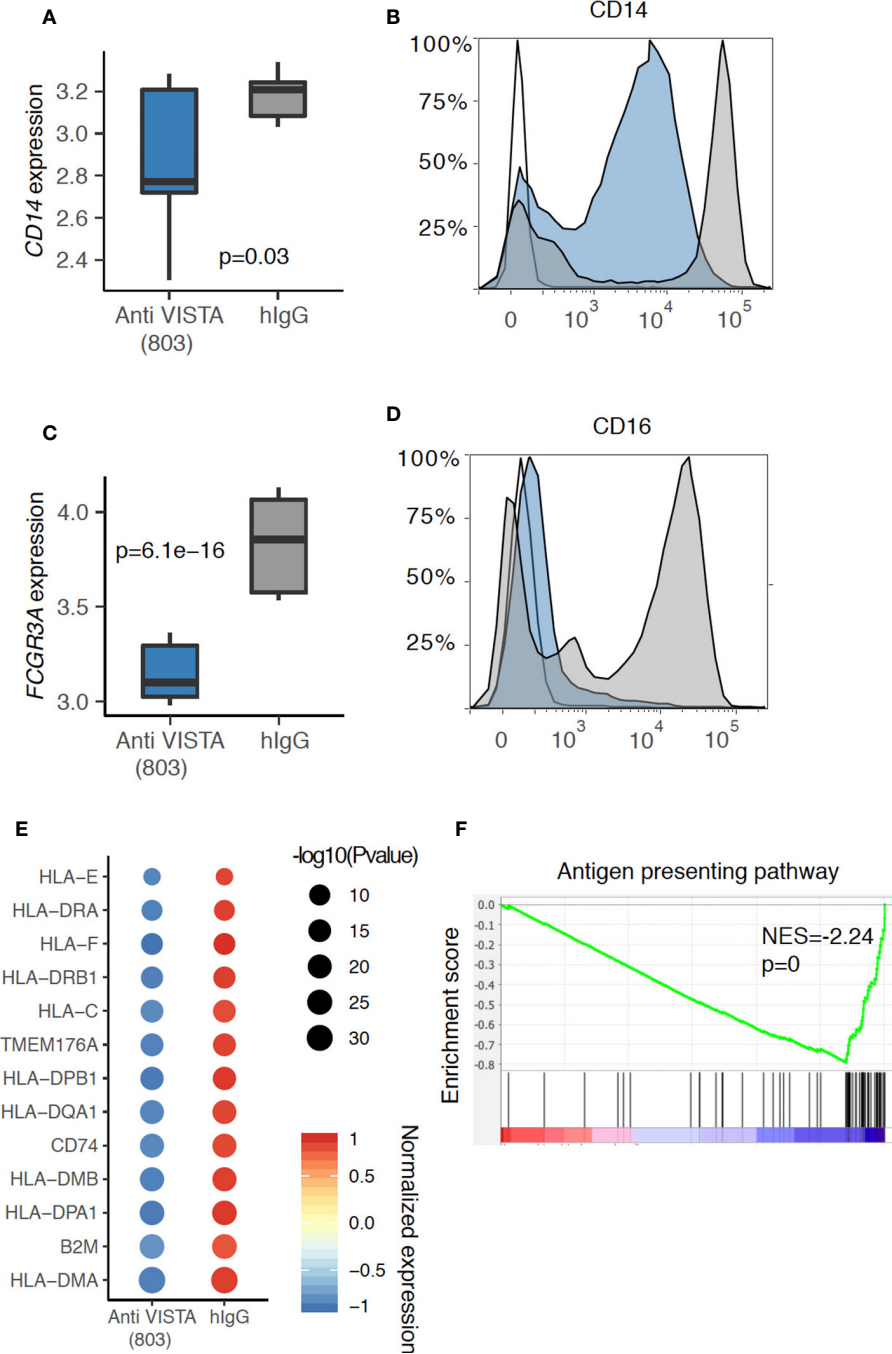
Anti-VISTA agonist suppresses CD14 and CD16 (*Fcgr3a*) expression in human monocytes. **(A)** Boxplot depicting the CD14 gene expression difference between Anti-VISTA (803) and hIgG2 isotype control treated CD14+ human monocytes. **(B)** Flow cytometry plot showing the CD14 protein expression between Anti-VISTA (803) and hIgG2 isotype control treated CD14^+^ human monocytes. **(C)** Boxplot depicting the *Fcgr3a* gene expression difference between Anti-VISTA (803) and hIgG2 treated CD14+ human monocytes. **(D)** Flow cytometry plot showing the CD16 protein expression between Anti-VISTA (803) and hIgG2 treated CD14+ human monocytes. Flow cytometry experiments are representative of three independent experiments with one donor per each experiment. **(E)** Dot plot showing the antigen presenting associated genes difference between Anti-VISTA (803) and hIgG2 isotype control treated CD14+ human monocytes. **(F)** GSEA plot showing the enrichment of antigen presenting pathway between Anti-VISTA (803) and hIgG2 treated CD14+ human monocytes. RNA-seq experiments are representative of three independent repeats on three healthy donors.

While the groundwork for a central role of VISTA in controlling T cell biology has been created, emerging data show an equally important and global role of VISTA in controlling innate inflammation. Studies discussed define a significant role for VISTA in controlling myeloid chemotaxis, antigen presentation, and fate determination. Unique to VISTA, as an NCR, is its role in the regulation of chemotaxis. It was shown that the genetic loss of VISTA reduced the expression of C5aR1 on monocytes and macrophages and inhibited their migration to the cognate chemoattractant ligand C5a (15). A subsequent study revealed that the regulatory impact of VISTA was not limited to the C5a/C5ar1 axis, but exerted a broad impact on the expression of several chemokines and chemokine receptors (25). VISTA deficiency and antibody targeting was shown to reduce CCR2 and CX_3_CR1 expression on monocytes; two hallmark receptors for Classical and Patrolling murine monocytes, respectively (25). Of note, VISTA targeting also strikingly reduces CD14 and CD16 (FcγIIIa) expression; again defining receptors for Classical and Patrolling human monocytes, respectively as shown by flow cytometry and RNA-seq analyses (**Figures 1A–D**). In addition, loss of VISTA enhanced the levels of the chemokines CCL2, CCL3, CCL4, and CCL5 by macrophages at steady-state (25). The authors attributed this enhancement to reduced consumption of these chemokines by VISTA-deficient macrophages owing to reduced steady-state CCR2 expression and enhanced CCR5 downregulation in response to their cognate chemokines. As a result, these cells had profound chemotactic deficits in the responses towards these chemokines. Very recent work also demonstrated an impact of VISTA targeting on reducing CXCR2 expression on neutrophils, with the virtual ablation of their migratory responses to the CXCR2 ligand (CXCL2) *in vitro* and *in vivo* (Li et al., personal communication). These results highlight VISTA as an important checkpoint that regulates the response towards multiple chemokine/chemokine receptor networks. The migratory response of immune cells represents the earliest checkpoint towards inflammatory stimuli. It is tempting to suggest that interfering with this pathway can eliminate or modulate immune responses prior to their exacerbation. These intriguing results also present the prospect of VISTA targeting being crucial for regulating myeloid cell responses in the context of inflammatory diseases where neutrophils and monocytes play dominant roles.

Accumulating evidence from multiple systems suggests that VISTA may play a role in the regulation of antigen presentation cell (APC) activity. At the level of expression, VISTA has been reported to colocalize with MHC-II, and VISTA overexpression in myeloid cells reduced MHC-II expression levels (26). In a melanoma tumor model, VISTA blockade enhanced the activation state of conventional dendritic cells (cDCs), upregulating the expression of MHC-II and CD80, as well as augmenting the production of IL-12 and TNFα (27). In contrast, studies with VISTA agonists has revealed that agonist treatment of human monocytes induced a profound and broad time-dependent downregulation of MHC-II genes as well as CD80 (**Figure 1E**). This is also supported by pathway analysis where the antigen presentation pathway was significantly downregulated in anti-VISTA agonist treated monocytes (**Figure 1F**). Our recent findings also demonstrate that VISTA agonist suppresses IL-12 production from myeloid cells under LPS stimulation conditions (28). Therefore, published studies and studies presented herein are providing documentation that VISTA plays an early and central role in the control myeloid migration and antigen presentation. In this context, VISTA is a primary target for controlling the earliest phases of innate inflammation.

VISTAis an immunoregulatory factor regulating myeloid fate determination. Loss of VISTA exacerbated psoriasis and the investigators attributed this effect [in part] to enhanced TLR7 signaling on DCs. More recent mechanistic insights into VISTA regulation of myeloid biology revealed a role for VISTA in modulating the ubiquitination and expression of the TLR-MyD88 effector TRAF6, and by consequence, the negative regulation of TLR signaling and the downstream MAPK and NFkB axes (29). As a result, loss of VISTA on macrophages enhanced cytokine responses toward multiple TLR agonists including TLR2, TLR3, TLR4, TLR7, and TLR9. This agrees with our recent work showing that VISTA^-/-^ macrophages showed enhanced cytokine responses to TLR4 stimulation (25). As one would anticipate, overexpression of VISTA in a monocytic-cell line (THP-1) dampened responses to TLR2 stimulation (29). To gain a global perspective of the impact of VISTA targeting on myeloid fate, a more comprehensive assessment of transcriptional reprogramming by VISTA on human monocyte transcriptome was performed and is presented.

## VISTA Induce Myeloid Reprogramming: Evidence for Profound Reprogramming and a Target in COVID Cytokine Storm Management

**Figure 2:**
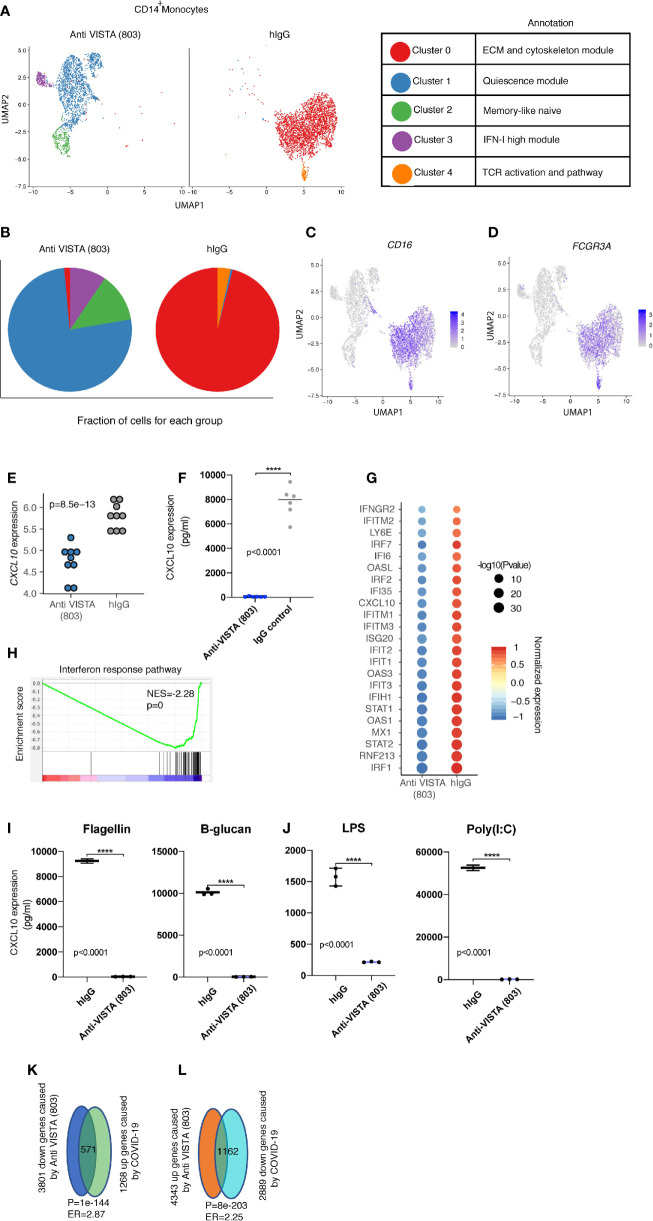
Anti-VISTA agonist strikingly changes the CD14^+^ monocyte state and induces novel archetypes associated with the anti-inflammatory phenotype. **(A)** Uniform manifold approximation and projection (UMAP) plot showing the cluster distribution of Anti-VISTA (803) and hIgG2 isotype control treated CD14^+^ human monocytes. The biological annotation of each cluster is presented in the table on the right. **(B)** Pie chart indicating the composition of cluster difference in Anit-VISTA (803) and hIgG treated CD14+ human monocytes. **(C, D)** Feature plot showing the expression of *Cd14* and *Fcgr3a* across different clusters in Anti-VISTA (803) and hIgG treated CD14^+^ human monocytes. **(E)** Dot plot depicting the CD14 gene expression difference between Anti-VISTA (803) and hIgG2 isotype control treated CD14^+^ human monocytes. **(F)** Human CD14^+^ monocytes were either treated with anti-VISTA agonist or hIgG2 isotype control for 24 h and CXCL10 supernatant levels was determined *via* multiplex analysis **(G)** Dot plot showing the interferon response associated genes difference between Anti-VISTA (803) and hIgG2 isotype treated CD14^+^ human monocytes. **(H)** GSEA plot showing the enrichment of interferon response pathway between Anti-VISTA (803) and hIgG2 treated CD14+ human monocytes. **(I, J)** Supernatant levels of CXCL10 determined by multiplex analysis after anti-VISTA agonist or control hIgG2 treatment of monocytes in the presence of Flagellin, B-glucan, LPS or Poly(I:C). **(K, L)** The Venn diagram showing the significant enrichment between Anti-VISTA (803) treated and COVID-19 CD14+ human monocytes. These experiments are representative of three independent repeat. ****p < 0.0001.

Single-cell RNA-seq of anti-VISTA agonist treated human monocytes revealed a profound shift and almost complete elimination of the CD14^+^ classical monocyte phenotype in favor of a more anti-inflammatory cell state characterized by a striking downregulation of CD14, IFN receptors, Fcγr3a (CD16), and CSF1R. In parallel, the major VISTA-induced cluster 1 upregulated CD11b, M-CSF (Csf1), Cyclin-dependent kinase inhibitor (Cdkn1a), the Src kinase inhibitor *Matk*, and the anti-inflammatory cytokines IL1RA and GDF15 (**Figures 2A, B**). Cdkn1a has been reported to suppress arthritis in mouse models and reduced monocyte inflammation (30, 31), whereas GDF15 and IL1RA have documented roles as critical inhibitors of LPS responses, septic shock, and inflammatory responses in several disorders (32–37). There were three other cell states (clusters) specifically induced by anti-VISTA but these present a minority of the total cells, therefore we will not discuss them in detail. In agreement with the flow cytometry data on anti-VISTA agonist treated monocytes (**Figure 1**), a hallmark of the VISTA agonist induced monocyte cell state was a near-complete downregulation of CD14 and CD16 (**Figures 2C, D**). Additional analysis of the anti-VISTA agonist impact on CD14^+^ human monocytes at steady-state (unactivated) revealed a complete suppression of CXCL10 production, even in the presence of potent stimulatory pattern recognition ligands (PRRs) (**Figure 2**), reproducible across heterogeneous donors. This suppression was manifested at the transcriptional (**Figure 2E**) and proteomic levels (**Figures 2E, F, I, J**). We argue that the suppression of CXCL10 is a consequence of a penetrant downregulation of the IFN-I pathway genes, including its upstream effector STAT1 (**Figures 2G, H**). Even after stimulation with multiple pattern recognition receptor (PRR) ligands, the anti-VISTA suppression of CXCL10 was maintained (**Figures 2I, J**).

Recent reports highlight CXCL10 as a prognostic biomarker and critical pathogenic mediator of COVID-19. Given the pronounced impact of anti-VISTA on suppression of CXCL10, these findings ultimately lead to a potential immunoregulatory role of VISTA in COVID-induced inflammation and pathology. Despite the recovery of most infected individuals, a significant number of COVID-19 patients present with severe respiratory distress in addition to complications including a hyperinflammatory response (38, 39). Several studies pointed towards the central contribution of the mononuclear phagocyte compartment to this hyperinflammatory cytokine production associated with disease immunopathology (6). Recent immunophenotyping analysis of the peripheral blood from a large heterogeneous pool of COVID-19 patients reveal a core consensus immune signature (40). Within this signature, sustained overexpression of the IFN-I/II inducible chemokine CXCL10 had a striking positive correlation with evolving disease severity, and was the most reliable prognostic biomarker. This immune signature highlighting CXCL10 chronic upregulation was further supported by two independent studies (41, 42). It is worth noting that CXCL10 was also highly upregulated with other coronaviruses SARS1 (43) and MERS (44–46), also positively correlating with disease severity. As in human studies (40, 42), novel mouse models of SARS-CoV-2 also show that type I IFN does not control viral infection but is a driver of COVID immunopathology (47). Enrichment analysis to the COVID-19 immune profile revealed that anti-VISTA agonist downregulated over 40% of the hallmark genes defining the COVID-19 immune signature (**Figures 2K, L**). This indicates that VISTA agonist may suppress the COVID19 inflammatory signature. Therefore, VISTA intersects with the CXCL10 induction pathway which is of relevance to COVID-19 immunopathology. It is also critical to highlight that the reduction of FcγRIIIa expression by VISTA targeting is of significant interest as hyperinflammatory Fc receptor responses have been reported as an immunopathologic manifestation of COVID-19 infection (48).

Beyond the striking impact on transcriptional reprogramming exerted by VISTA, the impact of VISTA on myeloid chemotaxis may also play an important therapeutic role in controlling innate inflammation in COVID. The high neutrophil to lymphocyte ratio in critically ill COVID-19 patients has been predictive in hospital mortality (49). Recent reports and commentaries implicate neutrophils as critical components of the hyperinflammatory responses to COVID-19, and suggest that impeding neutrophil recruitment *via* CXCR2 may be a promising treatment in this setting (50, 51). Our recent analysis of anti-VISTA agonist impact on neutrophil biology demonstrates a clear suppression of CXCR2 expression, and by consequence, their migratory responses, in both murine and human neutrophils. This would indicate a potential mechanism whereby VISTA targeting could suppress neutrophil chemotaxis and shut down the inflammatory circuit. Therefore, we argue that VISTA agonists may be of valuable therapeutic relevance in a broad spectrum of inflammatory settings.

Could VISTA agonists provide a valuable intervention tool to ameliorate the fatal cytokine-release syndrome (CRS) induced by CAR-T cell therapy in certain patients? Based on our T cell studies, VISTA agonists likely would exert minimal inhibitory impact on activated CAR T cells directly. However, we understand that CAR T cells activate macrophages or neutrophils to cause organ damage and other adverse events (52). As a result, heightened cytokine production from myeloid cells likely contribute to CRS and agonistic targeting of VISTA may ameliorate the innate components of the CAR T induced CRS.

After 10 years of the first report on VISTA as an inhibitory receptor of immune responses, there remains yet an absence of any primary studies or perspectives on the role of this molecule in the settings of immune response to infection. Given the imminence of the COVID-19 viral infection pandemic and the lack of knowledge on the etiologies behind the unbalanced immune responses and pathophysiology that account for its severity, observations reported herein offer some insights on how VISTA targeting could be utilized to normalize innate and adaptive immune responses in these pathologic settings.

## The VISTA Ligand: Current State of the Science

A key challenge against the development and understanding of anti-VISTA targeting strategies is the absence of knowledge with regards to a VISTA ligand [or counter-receptor]. Despite over 40 published studies by VISTA thought-leaders (the Noelle and Chen groups), no reports included any insight into potential VISTA ligands, despite the experience of both groups in identifying ligand receptor pairs. This truly highlights the difficulty of identifying an endogenous functionally-relevant ligand for VISTA. However, recent studies have presented several possible candidates. VSIG3 (also named IgSF11) has been identified a major ligand for VISTA demonstrating specific binding and functional *in vitro* inhibition of T cell activation (53). Of interest are the overlapping binding region of VSIG3 and anti-human VISTA antagonist antibody on VISTA (54). Despite this, the undetectable expression of VSIG3 in the hematopoietic system [and indeed on all peripheral cells with the exception of reproductive tissue], several questions over its potential *in vivo* functional relevance to inhibition of immune cells *via* VISTA remain to be addressed. This does not exclude the possibility of relevance in tumor settings where VSIG3 could be expressed (55).

Another group reported the pH-dependent binding of VISTA to P-selectin glycoprotein ligand-1 (PSGL-1) (56). This study emphasized the importance of a unique histidine-rich region on the VISTA extracellular domain, wherein the histidine residue side-chains are protonated under acidic conditions which mediates VISTA binding to PSGL-1. In addition, tyrosine sulfation of PSGL-1 is also key to this binding. Of note, this binding epitope on VISTA is distinct from the epitope reported for VSIG3 and anti-VISTA binding (54). This extraordinarily high histidine content in VISTA and the consequential low pH dependent binding are of both conceptual and translational importance. However, there are several remaining avenues for investigation prior to confirming the relevance of this interaction to VISTA biology. First, no study has yet demonstrated any *in vivo* endogenous binding or interaction between VISTA and PSGL-1. Second, numerous studies presented several activities for VISTA on both T cells and myeloid cells under conditions where the pH environment was not changed. An interesting possibility is that VISTA : PSGL-1 pH-dependent interactions may occur in early and recycling endosomes where both molecules are highly expressed, and where the pH environment is indeed acidic (pH between 5.9 and 6.5) (57, 58), which ensures VISTA extracellular domain (now facing the lumen) protonation.

The concept of different signaling pathways mediated by an immune receptors depending on its location has been previously described, most famously for TLR-4 which triggers independent pathways at the plasma membrane versus the endosome (59). Both molecules are also highly expressed in macrophages, granulocytes and platelet, suggesting a potential role in these subsets, and indeed regulate stages of migration. However, ligands for endosomal or plasma membrane VISTA have not been conclusively demonstrated. It is of interest to note that the lymph node paracortical zones (where CD4^+^ T cells are enriched) are profoundly acidic, and this acidity is T-cell dependent whereby T cells acidity is a self-regulatory feedback mechanism to inhibit glycolytic rate and suppress the effector T cell response (60). Whether the acidic environment in certain tissues plays a role in VISTA signaling or function remains unclear.

A third VISTA ligand was recently identified to be matrix metalloproteinase 13 (MMP13) (61). A pull-down assay with MMP-13 with bone marrow cell lysates revealed enrichment of VISTA as a major binding protein. This binding was further confirmed by co-expressing the proteins in a cell-line and coimmunoprecipitation, and the VISTA extracellular domain was necessary for binding as revealed by mutagenesis studies. The authors argued that VISTA is the receptor for MMP-13 on osteoclasts, and that this signaling axis is relevant for osteoclast fusion and bone resorption in multiple myeloma. The expression of MMP13 within the hematopoietic compartment is mostly contributed by macrophages, especially in atherosclerotic lesions (62, 63), although a study pointed to a role in promoting DC activity (64). However, it is indeed also possible that VISTA could be one of the numerous targets for MMP-13 mediated cleavage (which include collagen and TNF) (65, 66).

## Conclusion

This work summarizes the most recent findings on the role of VISTA agonists in myeloid cell biology. This class of antibodies can directly elicit profound immunomodulatory effects on the myeloid subsets monocytes, macrophages and neutrophils even in the absence [and prior to] inflammatory stimulation. The broad impact of VISTA on these cells ranges from regulation of chemotactic responses, to the regulation of TLR signaling and the IFN pathway. There remain numerous avenues for future investigation; most importantly with regards to identification of the endogenous VISTA ligand(s) in addition to insights on its signaling roles to mediate these profound anti-inflammatory effects. Nevertheless, there are potential valuable therapeutic implications in the settings of dysregulated inflammation driven by innate cells which could instruct novel strategies in the treatment of autoimmunity and viral immunopathology.

## Author Contributions

Conceptualization: RN and ME. Methodology: RN, ME, YZ, ES, and CC. Investigation: ME, YZ, ES, and RM. Review and editing: RN, ME, YZ, CC, and RM. All authors contributed to the article and approved the submitted version.

## Funding

This work is supported by NIH grants R01AR070760 (RN), R01CA214062 (RN), and the Cancer Prevention Research Institute of Texas (CPRIT) (RR180061 to CC). CC is a CPRIT Scholar in Cancer Research.

## Conflict of Interest

RN is an inventor on patent applications (10035857, 9631018, 9217035, 8501915, 8465740, 8236304, and 8231872) submitted by Dartmouth College, and patent applications (9890215 and 9381244) submitted by Kings College London and Dartmouth College and a co-founder of ImmuNext, a company involved in the development of VISTA-related assets. These applications cover the use of VISTA targeting for modulation of the immune response.

The remaining authors declare that the research was conducted in the absence of any commercial or financial relationships that could be construed as a potential conflict of interest.
